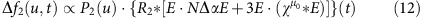# Erratum: Transient birefringence of liquids induced by terahertz electric-field torque on permanent molecular dipoles

**DOI:** 10.1038/ncomms15796

**Published:** 2017-05-25

**Authors:** Mohsen Sajadi, Martin Wolf, Tobias Kampfrath

Nature Communications 8: Article number: 14963; DOI: 10.1038/ncomms14963 (2017); Published: 04 10 2017; Updated: 05 25 2017

This Article contains typographical errors in six instances in which the convolution symbol 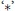 was mistakenly omitted from six equations during the production stage. Each instance is described below.

The penultimate sentence of the first paragraph of the Results subsection titled ‘Model' should read:

‘Therefore, Δ*f*_1_ does not yet cause optical birefringence but is accompanied by a time-dependent dielectric polarization 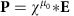.'

The correct form of equation (3) is:


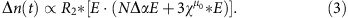


In the second paragraph after equation (3), the first sentence should read:

‘Note that equation (3) reveals an analogy of the **μ**_ind_- and **μ**_0_-related coupling mechanisms: the first field interaction generates an effective electronic (*N*Δ*α***E**) and orientational polarization (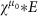) which, in turn, serves as a handle for the second field interaction to generate a *P*_2_-like perturbation (square bracket in equation (3)).'

In the paragraph preceding equation (11), the second sentence should read:

‘This polarization is usually expressed by the convolution 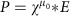 where 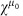 is the contribution of the permanent electric dipole moment **μ**_0_ to the familiar total dielectric susceptibility *χ*.'

The correct form of equation (11) is:


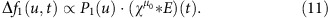


The correct form of equation (12) is: