# Supplementary material for: Transient birefringence of liquids induced by terahertz electric-field torque on permanent molecular dipoles
Source: Nat Commun. 2017 Apr 10;8:14963. doi: 10.1038/ncomms14963 (PMC5394237; doi:10.1038/ncomms14963)
Supplement: Supplementary Information — Supplementary Figures, Supplementary Notes and Supplementary References [file ncomms14963-s1.pdf]

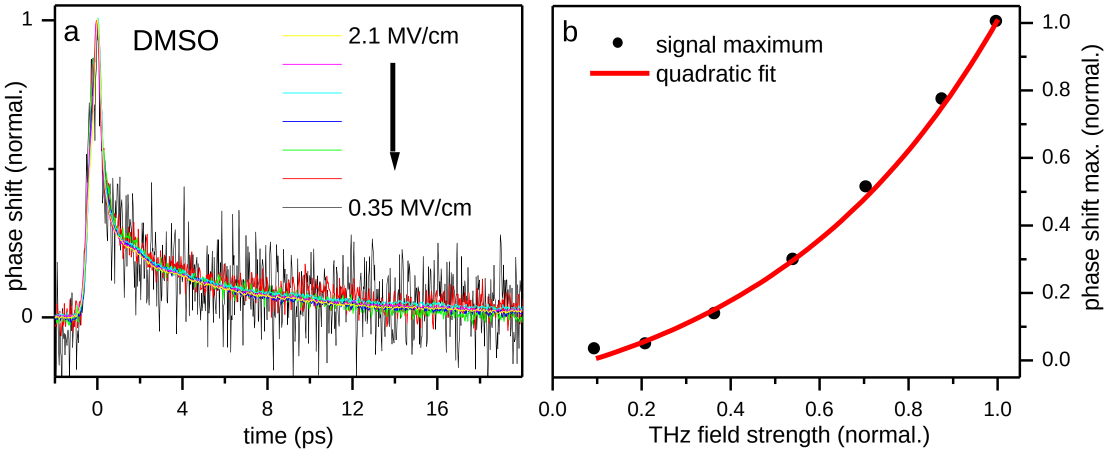

Supplementary Figure 1 | Pump-fluence dependence. a, THz Kerr signals of dimethyl sulfoxide (DMSO) measured with different pump fluences. Signals are normalized to the peaks of their electronic responses. **b**, Peak amplitudes of DMSO Kerr signals versus the peak THz field strength normalized to 2.1 MV cm^{-1}. The red solid line is a quadratic fit.

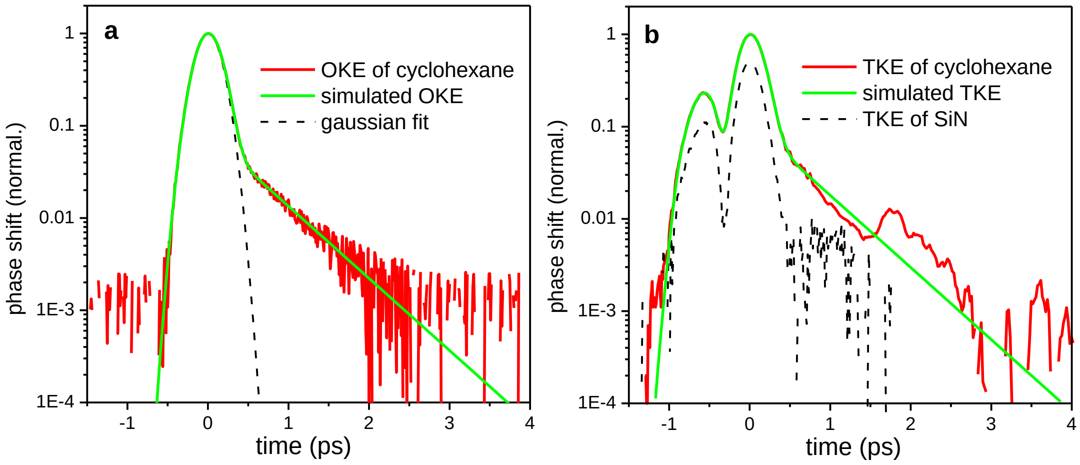

Supplementary Figure 2 | OKE and TKE convolution analysis. a, OKE signal of cyclohexane (red line) and its simulated signal according to Supplementary Eq. (1) (green line). The dashed line is a Gaussian fit to the left flank of the OKE signal. **b**, TKE signal of cyclohexane (red line) and its simulated signal (green line). For comparison, the dashed line shows the TKE response of a 200 nm thick SiN membrane.

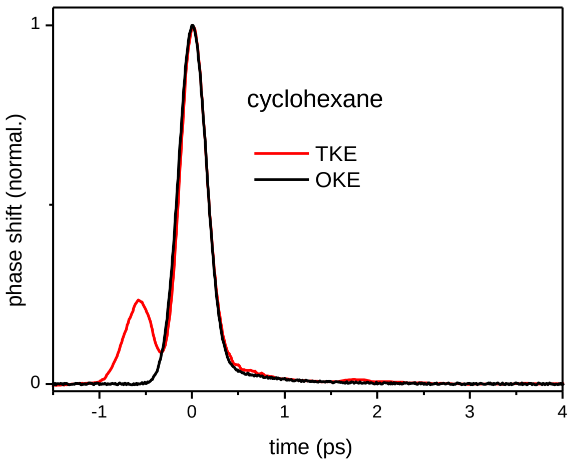

Supplementary Figure 3 | OKE vs TKE of cyclohexane. OKE and TKE signals of the nonpolar liquid cyclohexane, both normalized to the peaks of their electronic responses.

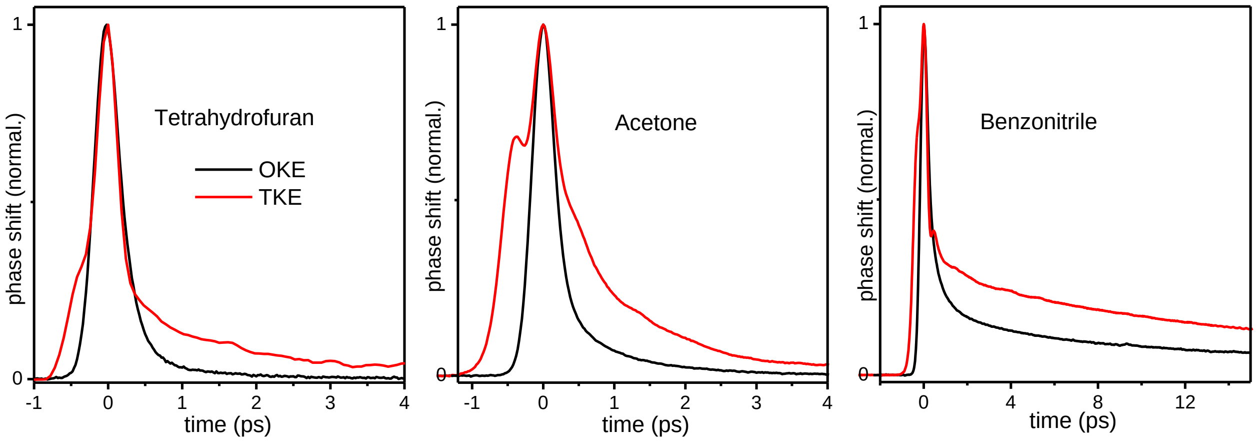

Supplementary Figure 4 | OKE vs TKE of polar liquids. TKE and OKE signals of three additional polar solvents with $\mu_0 > 0$ and $\Delta\alpha > 0$: tetrahydrofuran, acetone and benzonitrile. Signals are normalized to the peaks of their electronic responses.

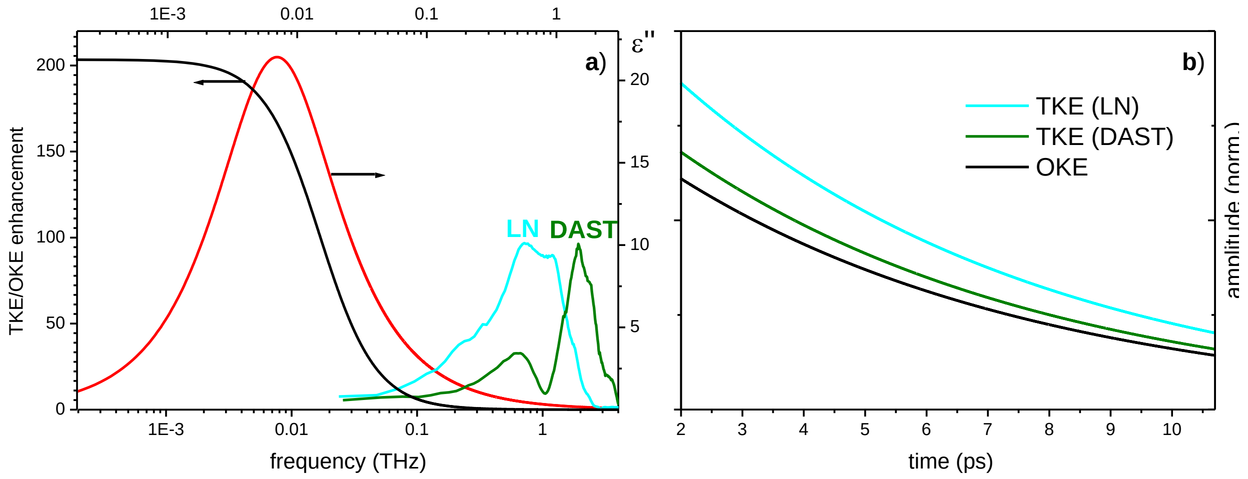

Supplementary Figure 5 | Predicted enhancement of TKE vs OKE amplitude. a, Estimated enhancement of TKE vs OKE signals of DMSO (black curve) as a function of the frequency of narrowband pump pulses, calculated by using Eq. (3) of the main text. The χ^{μ_0} is assumed to solely arise from Debye-like rotational relaxation with a time constant of 21 ps (imaginary part shown by the red curve). Cyan and green curves show the Fourier spectra of THz excitation pulses from LN and DAST sources, respectively. **b**, Simulated TKE and OKE signals, calculated by the same model as in panel **a**, but for the broadband THz pump pulses from the LN and DAST as well as the optical pump.

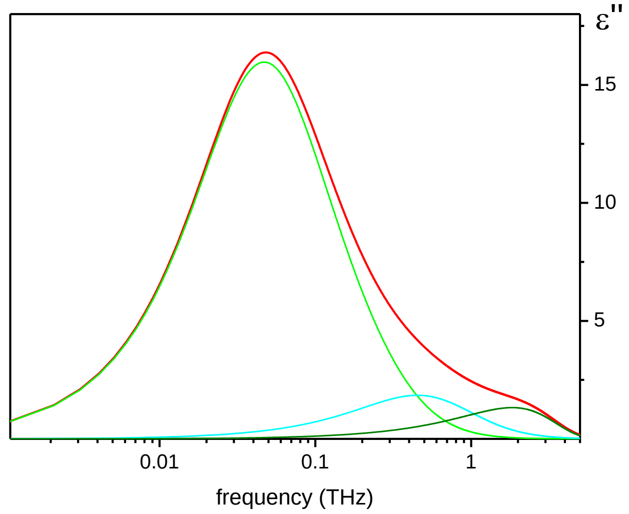

Supplementary Figure 6 | Dielectric function of acetonitrile. Dielectric loss spectrum of acetonitrile (red curve) with underlying Debye mode (green), translational β mode (cyan curve) and librations (olive curve)[1].

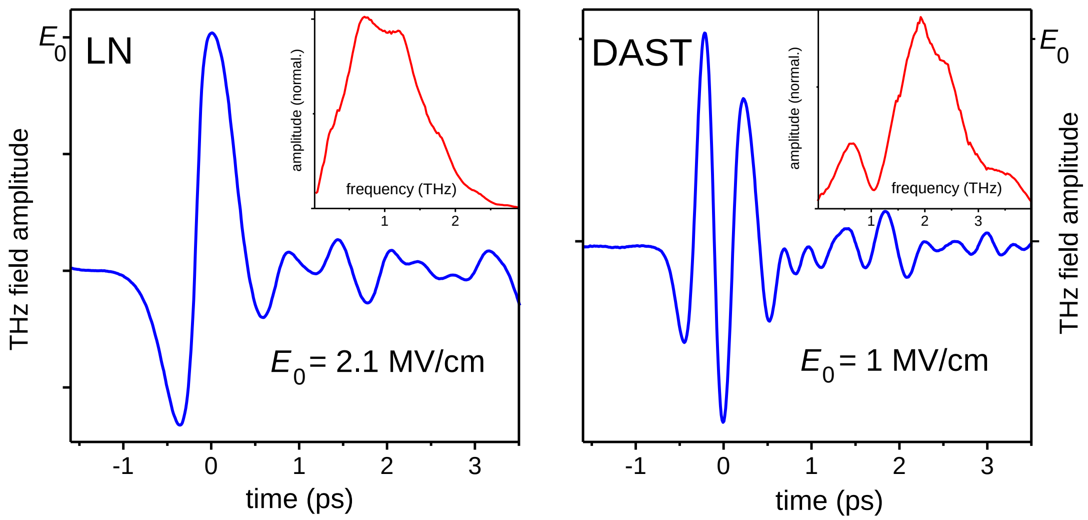

Supplementary Figure 7 | LN and DAST THz fields. THz electric fields from the LN and the DAST nonlinear optical crystals. The corresponding Fourier spectra are shown in the insets.

Supplementary Note 1: Optical vs THz Kerr effect of cyclohexane.

Here, we provide more detailed support of the notion that the dynamics Kerr response of nonpolar liquids is independent of excitation frequency (optical or THz). To this end, we compare the optical Kerr effect (OKE) and the THz Kerr effect (TKE) dynamics of cyclohexane. We choose this liquid because (i) its permanent molecular dipole moment is zero ($\mu_0 = 0$), (ii) it is transparent at both optical and THz pump-pulse frequencies, and (iii) the velocity mismatch of THz and optical pulses in cyclohexane is small[2]. The measured OKE and TKE signals are, respectively, shown in Supplementary Figs. 2a and 2b (red lines) along with the intensity profile $I_{\text{pu}}(t)$ of the optical and THz pump pulses (dashed lines).

In a pump-transparent medium, the dynamic Kerr signal is phenomenologically given by[3]

$$S(t) = [A\delta(t) + R_{\text{nuc}}(t)] * I_{\text{pu}}(t) \quad (1)$$

where the first and second term, respectively, quantify the instantaneous electronic response (described by the constant A) and the relaxation of the nuclear degrees of freedom (described by the response function $R_{\text{nuc}}(t)$). For the OKE response (Supplementary Fig. 2a), we follow Ref. 4 and estimate the intensity envelope of the pump pulse by fitting a Gaussian function to the left flank of the OKE signal (dashed line in Supplementary Fig. 2a). Once $I_{\text{pu}}(t)$ is determined, we fit Supplementary Eq. (1) to the OKE signal where $R_{\text{nuc}}(t)$ is assumed to be a monoexponentially decaying step function. The agreement of data and fit (green line in Supplementary Fig. 2a) is excellent.

To fit the TKE data (Supplementary Fig. 2b), we first determine $I_{\text{pu}}(t)$ by measuring the TKE signal of an empty cuvette (dashed line in Supplementary Fig. 2b). The response of the cuvette SiN window (thickness of 200 nm) is more than two orders of magnitude smaller than the liquid response and, therefore, neglected in the following. According to Supplementary Eq. (1), we convolute $I_{\text{pu}}(t)$ by the same exponential relaxation curve obtained from fitting of the OKE signal. As seen from Supplementary Fig. 2b, the calculated (green line) and measured (red line) signals agree excellently.

Therefore, the dynamic Kerr response $A\delta(t) + R_{\text{nuc}}(t)$ of nonpolar liquids is identical for optical and THz excitation, apart from a global scaling factor. Note that this factor is one to a very good approximation because THz (<10 meV) and optical (~1.5 eV) pump photon energies are much smaller than the electronic excitation energies (>5 eV) of cyclohexane[5]. Therefore, A (which quantifies the instantaneous electronic response) is equal at THz and optical frequencies, and so is the complete Kerr response, including $R_{\text{nuc}}(t)$.

We finally compare the OKE and TKE signals of cyclohexane directly and normalize to the respective maxima of their electronic responses. Interestingly, as shown in Supplementary Fig. 3, both signals are almost identical. This behavior arises because both THz and optical pulses have comparable temporal duration of ~350 fs. Therefore, to compare the amplitude of the nuclear contribution to the pump-induced transient birefringence for a given pump-pulse energy in our experiment, it is sufficient to normalize the pump-probe signal to the maximum of their initial electronic response.

Supplementary Note 2: Model calculation of birefringence enhancement.

To illustrate the model developed here (see 'Methods' section), we calculate the enhancement factor of the induced birefringence following THz vs optical excitation of DMSO by using Eq. (3) (main text) and assuming monochromatic excitation. In addition, the χ^{μ_0} (the $\boldsymbol{\mu}_0\times\mathbf{E}$-related component of the dielectric susceptibility) is assumed to solely arise from Debye-like rotational relaxation with a time constant of 21 ps, resulting in maximum absorption (and thus maximum $\mathrm{Im}\,\chi^{\mu_0}$) at ~7 GHz (red line in Supplementary Fig. 5a). Interestingly, maximum enhancement is achieved at even lower frequencies (black line in Supplementary Fig. 5a). At a pump frequency of ~1 THz, enhancement is close to zero, in agreement with previous work[6,7].

We note that the frequency-dependent enhancement factor of Supplementary Fig. 5a serves to illustrate the impact of increasing pump frequency. It is directly applicable to pump pulses with very narrow spectrum, whereas for the very broadband pulses from the DAST and LN sources used in our experiments, we should use Eq. (3) (main text) together with the transient electric field of the THz pump pulse (see Supplementary Fig. 7). The transient birefringence for the DAST and LN pump pulses is shown in Supplementary Fig. 5b. As expected from the trend of Supplementary Fig. 5a, increased birefringence amplitude is obtained for the LN pump, whose spectrum is centered at lower frequencies (~1 THz) than the DAST spectrum (~2 THz, see Supplementary Figs. 5a and 7).

Supplementary References

1. Stoppa, A., Nazet, A., Buchner, R., Thoman A. & Walther, M. Dielectric response and collective dynamics of acetonitrile. *J. Mol. Liq*. **212**, 963–968 (2015).
2. Pedersen, J.E. & Keiding, S.R. THz time-domain spectroscopy of nonpolar liquids. *Ieee Journal of Quantum Electronics* **28**, 2518-2522 (1992).
3. McMorrow, D. & Lotshaw, W.T. Intermolecular dynamics in acetonitrile probed with femtosecond fourier-transform Raman-spectroscopy. *J. Phys. Chem.* **95**, 10395-10406 (1991)
4. Ernsting, N.P., Photiadis, G.M., Hennig, H. & Laurent, T. Rotational friction kernel in water from the femtosecond time-resolved optical Kerr effect of acetonitrile/water mixtures. *J. Phys. Chem. A,* **106**, 9159-9173 (2002).
5. Boyd, R.W. Nonlinear Optics (Academic, San Diego, Calif., 1992).
6. Häberle, U. & Diezemann, G. Dynamic Kerr effect responses in the terahertz range. *J. Chem. Phys.* **122**, 184517 (2005).
7. Häberle U. & Diezemann G. Kerr effect as a tool for the investigation of dynamic heterogeneities, *J. Chem. Phys.* **124**, 044501-8 (2006).
